# Raman and IR Spectroelectrochemical Methods as Tools to Analyze Conjugated Organic Compounds

**DOI:** 10.3791/56653

**Published:** 2018-10-12

**Authors:** Agata Blacha-Grzechnik, Krzysztof Karon, Przemyslaw Data

**Affiliations:** ^1^Faculty of Chemistry, Department of Physical Chemistry and Technology of Polymers, Silesian University of Technology; ^2^Department of Physics, Durham University; ^3^Centre of Polymer and Carbon Materials of the Polish Academy of Sciences

**Keywords:** Chemistry, Issue 140, Cyclic voltammetry, Raman, Infrared Spectroscopy, Organic Electronics, Conjugated Polymers, Spectroelectrochemistry, Attenuated Total Reflection

## Abstract

In the presented work, two spectroelectrochemical techniques are discussed as tools for the analysis of the structural changes occurring in the molecule on the vibrational level of energy. Raman and IR spectroelectrochemistry can be used for advanced characterization of the structural changes in the organic electroactive compounds. Here, the step-by-step analysis by means of Raman and IR spectroelectrochemistry is shown. Raman and IR spectroelectrochemical techniques provide complementary information about structural changes occurring during an electrochemical process, *i.e.* allows for the investigation of redox processes and their products. The examples of IR and Raman spectroelectrochemical analysis are presented, in which the products of the redox reactions, both in solution and solid state, are identified.

**Figure:**
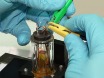


## Introduction

The combination of electrochemical and spectroscopic techniques allows for the possibility of tracking the structural changes in molecules present at the electrode surface or in the solution, thus investigating the mechanism of the electrochemical processes. Spectroelectrochemical methods are typically used for the *in situ* study of the mechanism of the reaction. The undoubted advantage over *ex situ* measurements is the possibility of observing the signal arising for the intermediate products of processes or investigating the processes, in which products cannot be separated^1^. Among all spectroscopies, the Raman and infrared spectroscopies are the most powerful for analysis of electrochemical processes due to equipment availability and the often non-destructive nature of the measurements.

Infrared and Raman spectroscopies provide information about the vibrational structure of the species and thus the existing chemical bonds. Since the nature of the signals observed in both techniques is different, some vibrations may be active only in IR or Raman spectra, making them complementary to each other^2^. This should be taken into account, when planning spectroelectrochemical analysis and, if possible, the vibrational structure of an analyte should be examined using both IR and Raman spectroscopies. The best results are obtained when the changes in the structure are the result of the electrochemical process involving groups active in the certain technique. For example, the infrared spectroscopy would be ideal for processes involving -CO, -CN -NO or -NH groups' formation or breakage^3^. It is always recommended to register differential spectra of the spectroelectrochemical investigation. Also, such spectra disclose changes in the signals with lower intensity allowing the tracking of changes in the structure of the aromatic systems. Additionally, differential spectra are always less complex as only changes are registered, which makes the interpretation of the spectra much easier.

IR spectroelectrochemical experiments are mainly used for the monitoring of the soluble products, intermediates and reactants of the electrochemical reactions; such tests may be run on various systems, including organic, inorganic, or biochemical systems^3^,^4^,^5^,^6^,^7^,^8^. One should always remember that in the case of IR spectroscopy, solvents in which hydrogen bonding occurs, like water, should be avoided.

There are several ways to proceed with IR and Raman measurements. In the case of IR spectroscopy, measurements can be done in the transmission mode, in which conventional IR cuvettes for liquids can be used. The optically transparent electrodes (*e.g.,* boron-doped diamond electrode) or perforated electrodes (metal gauze working electrode) made of fine metal (Pt or Au) are usually used as the working electrodes in such transmission cells^4^,^9^. An example of the transmission spectroelectrochemical cell is presented in the **Figure 1**.

In the second technique, instead of transmission, the reflectance mode is applied, thanks to the ATR (Attenuated Total Reflection) attachment^10^. This method allows analyzing both solutions and solid-state materials. Typically when using the method of external reflection absorption spectroscopy, in principle, any working electrode can be used, but only dissolved species can be investigated. However, in some cases, the ATR technique allows also for the investigation of processes in the solid state, using the internal reflection method^5^,^8^. A special cell is required for this technique, in which the fine metal sputtered on the ATR crystal acts as a working electrode (**Figure 2**). In some cases, even the ATR Ge crystal itself can act as an electrode (at least for not too high currents)^5^.

The second technique is Raman spectroelectrochemistry; a technique combining both electrochemistry and Raman spectroscopy, commonly used in the investigation of the potentially-induced structural changes in the deposited layer of conjugated polymers^11^, like polyaniline^12^, polypyrroles^13^, polycarbazole^14^ or PEDOT^15^. Additionally to polymeric films, monolayers can be also tested^19^,^20^,^21^, though in this case metallic substrates, like gold or platinum, are preferred. The procedure of Raman spectroelectrochemical studies is analogical to other spectroelectrochemical techniques, *i.e.,* a spectrometer must be coupled with a potentiostat and the spectra of the film are acquired in the potentiostatic conditions under various potentials' applied^18^. Typically, the three-electrode spectroelectrochemical cell can be constructed based on the classical quartz cuvette with electrodes mounted in a Teflon holder (**Figure 3**). The acquisition parameters, like the type of the laser, grating, *etc*., depend on the properties of the investigated layer. Selection of some parameters can be quite difficult, *e.g.,* one has to remember that various excitation wavelengths can result in different spectra. Usually, the higher energy of incident light the more details are visible on the spectrum, but also the higher risk of fluorescence phenomena that hinders the analysis. Generally, it is very useful to obtain the UV-Vis-NIR spectra of the analyte at first, in order to select the Raman excitation laser. The tunable lasers can be adjusted so that the excitation wavelength induces coincidence with an electronic transition of the molecule, resulting in the resonance Raman scattering. In this case, the increasing Raman scattering intensity in chosen regions of the spectra or even formation of new signals is observed that would not be registered typically. The analysis of the structural changes consists in the assignment of recorded Raman bands, which can be done based on the literature data or DFT simulations^23^.

## Protocol

### 1. Preparation of the Experiment

1. Cleaning procedure^24^ NOTE: The typical spectroelectrochemical cell consists of a platinum mesh(wire) or ITO working electrode (WE), Ag/AgCl or Ag electrode as a reference electrode (RE) and platinum coil or wire as an auxiliary electrode (AE) (Figure 1). All electrodes must be cleaned before use.
  1. Rinse a quartz ITO electrode with deionized water from the wash bottle. Then place it in an ultrasonic bath in a beaker filled with acetone for 15 minutes at room temperature and then in a beaker filled with isopropanol for the next 15 minutes. The higher ultrasound bath power that is used, the better result of cleaning.
  2. Let the electrode dry in the air.
  3. Burn platinum mesh or wire working electrode using a high-temperature torch (at least 500 °C) until it gets red (approximately 1 min). Remove it from the fire, when it turns red and then cool it in the air to room temperature (approximately 1 min). Be careful to not melt the mesh electrode.
  4. Polish the gold working electrode with emery paper (grit 2000) and then with 1 µm alumina. Ultrasonicate in deionized water for 5 minutes. Afterwards, rinse the electrode three times with the solvent used for the measurements. Use working electrodes directly after cleaning.
  5. Burn the active area of the auxiliary electrode (platinum wire or spiral) using a high-temperature gas torch (at least 500 °C) until it gets red (approximately 1 min) and then cool in the air to room temperature.
  6. Take the reference electrode out from the storage electrolyte and wash it three times with the solvent used for the measurements.
  7. Clean the spectroelectrochemical vessel with alcohol (ethanol or isopropanol) or acetone using a syringe and air dry. Clean all other elements (*i.e.*, Teflon parts) with acetone and air dry before use (minimum 1 minute).
2. Prepare at least 10 mL of the supporting electrolyte solution. The electrolyte solution should fulfil the same requirements as for standard electrochemical experiments, *i.e.,* its concentration should be at least 100 times higher than the concentration of the analyte. An example electrolyte could be 0.2 M solution of Bu_4_NPF_6_ in dry acetonitrile or dichloromethane and a sample concentration of 1 mM. Water solutions, like 1 M H_2_SO_4(aq)_, can be also used for Raman spectroscopy. If it is possible, use solvent and electrolyte of the highest purity.
3. In the case of spectroelectrochemistry of an analyte present in solution, prepare at least 1 mL of an analyte solution at 1 mM concentration in the electrolyte solution.
4. Put the argon (or nitrogen) Teflon pipe in the solution and start bubbling it for at least 5 min, in order to remove residual oxygen from the solution. Control the gas flow, so that only small bubbles appear at the solution surface. Do not use too high gas flow; otherwise, the solvent will evaporate from the vessel. In order to reduce solvent evaporation, a gas saturated with the solvent can be used. In this case, the inert gas must flow through a container with dry solvent before flowing to your electrolyte solution.

### 2. IR Spectroelectrochemistry

1. Run electrochemical (cyclic voltammetry) tests on the analyte before starting the spectroelectrochemical analysis^7^, so that the potential range, in which the redox processes occur, or the reversibility of the redox processes, can be determined.
2. Assemble the experimental cell equipped with three electrodes (working mesh platinum, auxiliary and reference) as presented in Figure 1. Ensure there is no leakage by filling it with the pure solvent. In the case of leakage, correct the mounting of the cell. When the cell is tight, remove the solvent with the syringe.
3. Turn on the IR spectrometer and the corresponding software.
4. Put the cuvette in the spectrometer holder. Fill the cuvette with the 2 mL of the analyte solution. The part of the working electrode, that will be irradiated with the incident beam, and the reference and auxiliary electrodes must be immersed in with the solution.
5. Connect the electrodes with corresponding wires going from the potentiostat using crocodile clamps. Ensure to not short circuit the electrodes (they should not touch each other).
6. Set the parameters of the spectra acquisition (spectrum range and resolution, the number of spectra to repeat in the case of FTIR). An example of typical parameters are as follows: spectrum range of 600-4000 cm^-1^, resolution of 1 cm^-1^, the number of spectra to repeat – 16.
7. Collect the IR spectrum by pressing the corresponding button on the spectrometer or in the software. Here, press the *Background* button to register a background and then press *Scan* button to collect spectrum.
8. Using the connected potentiostat apply a potential of 0.0 V to the working electrode and collect the IR spectrum (press the Scan button). Save the spectrum giving it an appropriate filename.
9. Change the applied potential, typically increase by a 100 mV, wait for app. 5 s, collect another IR spectrum and save it under the appropriate filename. Repeat this step until you reach entire potential range, in which oxidation/reduction processes may occur.
10. In order to check the reversibility of the structural changes during electrochemical oxidation or reduction, return to an initial potential (0.0 V) and collect the IR spectrum again. Either apply an increment of 100 mV or go straight back to the initial potential.
11. Subtract the initial spectrum from all other spectra to obtain differential spectra. Then, determine a straight line as the baseline by subtracting the neutral spectrum from itself in the software.
  1. From the menu **Process**, chose **Arithmetic**.
  2. In the newly opened window (**Arithmetic settings**), chose the operator from the dropdown list **Subtract (-)**.
  3. Select **Operand** and then select the spectrum registered at **0.0 V** from the dropdown list.
  4. To confirm the operation, press **OK**.
  5. To export data to a spreadsheet, choose **File | Send To | Excel**.
12. When finishing the spectroelectrochemical measurement, register a CV of the solution (refer to point 2.1 of the protocol). Then add the appropriate amount of ferrocene to receive its concentration of app 0.5 mM and register the CV again. NOTE: Ferrocene is used as a standard as its oxidation potential occurs at 4.8 eV. By recalculating the oxidation/reduction potentials of the sample vs. ferrocene, estimate the proper potential of the registered process and unify your data.

### 3. IR Spectroelectrochemistry in a Reflectance Mode

1. Perform the same measurement procedure as for the transmission mode. The only difference is in the cell assembly (2.4) which is different for each kind of experimental cell.

### 4. Raman Spectroelectrochemistry

1. Run the electrochemical (cyclic voltammetry) tests on the analyte before starting the spectroelectrochemical analysis^7^, so that the potential range, in which the redox processes occur, or the reversibility of the redox processes, can be determined.
2. Deposit the layer of interest on the wire or plate electrode by electrochemical polymerization or by dip casting method^12^.
3. Turn on the Raman spectrometer, laser and the corresponding software.
4. Put the experimental cell together. Place the three electrodes (working, reference and auxiliary) in the cuvette, so that they do not touch each other, as presented in the Figure 2. The best is to use a Teflon holder, well-fitting the cuvette.
  1. Place the working electrode (or working electrode covered with the deposited film) as close to the cuvette wall facing the incoming incident beam as possible, but do not press it to the wall (leave some space so the solution can flow easily between the cuvette wall and the working electrode).
5. Fill the cuvette with the electrolyte or analyte solution using a syringe (approximately 2 mL). Immerse all electrodes in the solution.
6. Put the cuvette into the holder in the Raman spectrometer and connect the electrodes with corresponding wires going from the potentiostat using crocodile clamps. Ensure that electrodes or connectors do not touch each other.
7. If the spectrometer is equipped with a camera, focus it onto the film deposited on the working electrode manually and/or using the software. A clear view of the working electrode surface should be visible.
8. Close the spectrometer's cover.
9. Choose the type of the laser desired and the corresponding grating based on the literature data or self-experience (for example the near infra-red 830 nm excitation laser and 1200 lines grating can be used). NOTE: The higher energy of the incident beam light the more accurate spectra can be obtained, but also the greater risk of fluorescence, which prevents spectrum analysis. The choice of the grating type will change the range and/or resolution of acquired spectra. Typically, the most appropriate laser and grating can be selected by testing all available.
10. Focus the laser beam on the working electrode surface using the software. If the spectrometer is equipped with a camera, the position at which the sharpest dot or line of the incident beam light on the sample is observed.
11. Set the parameters of the spectra acquisition: laser power, spectral range, time of illumination of the sample etc. The choice of the parameters depends on the type of the film or/and the substrate and should be selected individually. Do not use too high laser power; otherwise the sample is destroyed. Example parameters are as follows: laser power - 1%, spectra range - 400-3200 cm^-1^, time of illumination - 1 s, measurement repeated 3 times.
12. Collect the Raman spectrum by pressing the corresponding button on the spectrometer or in the software. If the intensity of the peaks on the registered spectra is weak, increase the power or acquisition time. A broadband in the spectra indicates the fluorescence phenomena. In this case, change the incident beam light to less energetic (higher wavelength) or try decreasing the laser power.
13. Apply a potential of 0.0 V to the working electrode using the connected potentiostat and collect the Raman spectrum. Save the spectrum giving it an appropriate filename.
14. Change the applied potential, typically increasing by 100 mV, wait for approximately 15 s, collect another spectrum and save it under the appropriate filename. Repeat this step until reaching the entire potential range where oxidation/reduction processes occur.
15. In order to check the reversibility of the structural changes during electrochemical oxidation or reduction, return to the initial potential (0 V) and collect Raman spectrum again.
16. When the spectroelectrochemical measurements are finished, register a CV of an analyte (present in solution or deposited on the electrode). Then, in the case of aprotic solutions, add the appropriate amount of ferrocene to obtain its concentration of app 0.5 mM and register the CV again. NOTE: Ferrocene is used as a standard, as its oxidation potential occurs at 4.8 eV. By recalculating the oxidation/reduction potentials of the sample vs. ferrocene, the proper potentials of registered processes can be determined.

## Representative Results

The structural changes of the monomer and polymer occurring during the doping are very useful to determine the mechanism of the process and for that, the IR spectroelectrochemical investigation can be conducted (Figure 4). In the example experiment, IR spectra were recorded in the differential form *i.e.* IR spectra of the investigated compound were taken as a reference. Such an approach allows for the exposure of the changes in the spectra occurring during polymerization: the disappearance of bonds are thus seen as a positive signal (increasing transmittance) while the formation of the new bonds is seen as negative peaks (decreasing transmittance) (Figure 4).

IR spectra recorded during analyte electropolymerization are shown in the Figure 4. As it can be seen, some changes occur at around 1600 cm^-1^, suggesting the disappearance of some of the material's double bonds. The most important are changes in the region between 700 - 900 cm^-1^: the increase in the transmittance at 750 and 675 cm^-1^ indicates the disappearance of the monosubstituted ring, simultaneously a new signal arising from the disubstituted ring appears around 830 cm^-1^. Based on the presented IR spectroelectrochemical experiment, the mechanism of the electropolymerization consisting in the reaction of the vinyl group with the free benzene ring is proposed.

In the presented example of Raman spectroelectrochemical studies, the potentially-induced structural changes of polyaniline film deposited on the electrografted layer of aniline (Figure 5) are investigated. The Raman spectra were recorded under potentiostatic conditions in the 1 M H_2_SO_4_ solution in the 800 - 1700 cm^-1^ range using 830 nm excitation laser and 1200 lines grating 25.

The Raman spectroelectrochemistry results of polyaniline electropolymerized on the electrografted gold substrate (*PANi/amino/Au*) are shown in the Figure 5. The signal assignment was based on the literature data^11^,^26^,^27^,^28^. At the starting potential of 0 mV, the bands at 1178 cm^-1^, 1265 cm^-1^ and 1608 cm^-1 ^arising from the C-H in-plane bending, C-N stretching, and C-C stretching respectively, are observed and confirm that the polyaniline below the potential of A-redox couple exists in the leucoemeraldine form. The increase in the applied potential above the potential of the first redox couple (A) causes the formation of the C-N stretching bands at 1239 cm^-1^ and 1264 cm^-1^, and semiquinone polyaniline structure that is indicated by two overlapping peaks within the 1300-1420 cm^-1^ region. Further increase in the potential up to 500-700 mV, *i.e.* above the potential of the second redox couple (B), causes a correlated growth of three bands: at 1235 cm^-1^- C-N stretching, at 1483 cm^-1^- C=N stretching and at 1590 cm^-1^- C=C stretching, which are characteristic for the deprotonated quinoid ring. This is accompanied by the decrease in the relative intensity of the 1335 cm^-1^ band, indicating the transition of polyaniline into the pernigraniline form.

**Figure:**
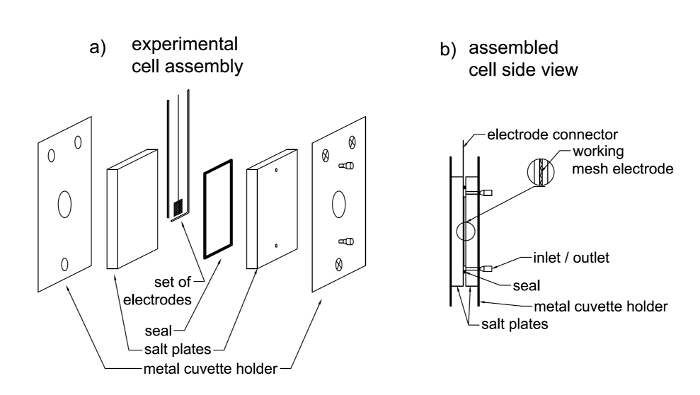

Figure 1 **: Scheme of transmission IR-spectroelectrochemistry cell (a) and its side view after assembly (b).** Please click here to view a larger version of this figure.

**Figure:**
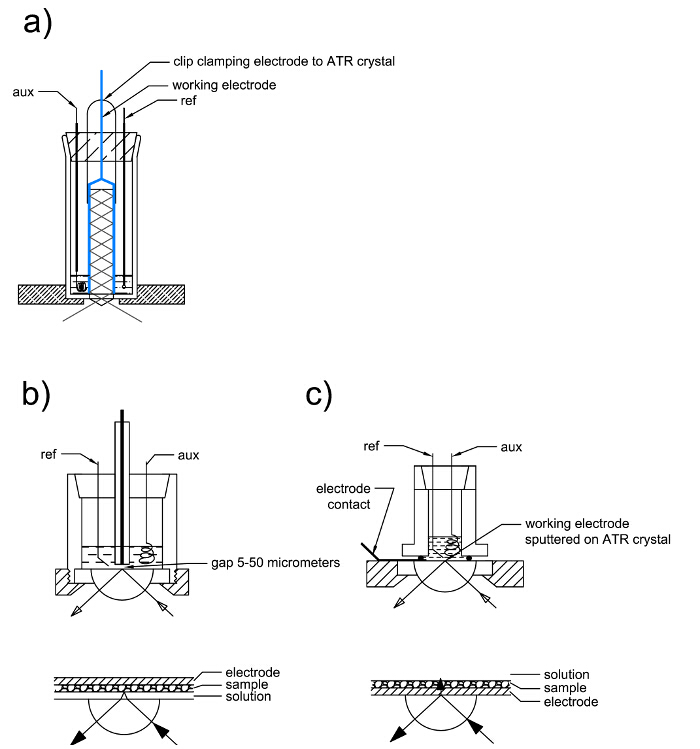

Figure 2 **: Schemes of reflectance cells for IR-spectroelectrochemistry. External reflectance cells a) and b) are used for the investigation of solute species. Internal reflectance cell c) is used for the investigation of species adsorbed on the electrode.** Please click here to view a larger version of this figure.

**Figure:**
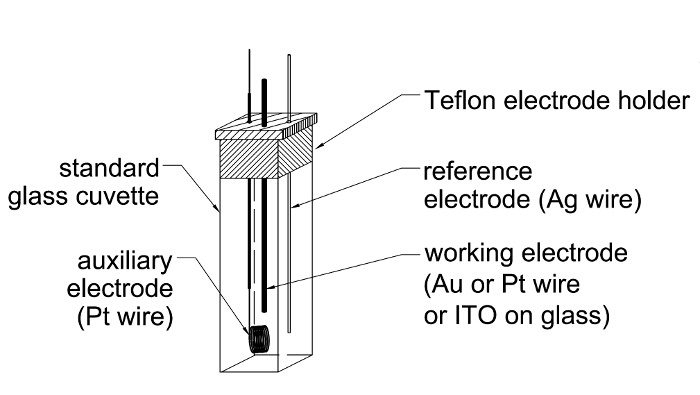

Figure 3 **: Schemes of Raman spectroelectrochemical cell** Please click here to view a larger version of this figure.

**Figure:**
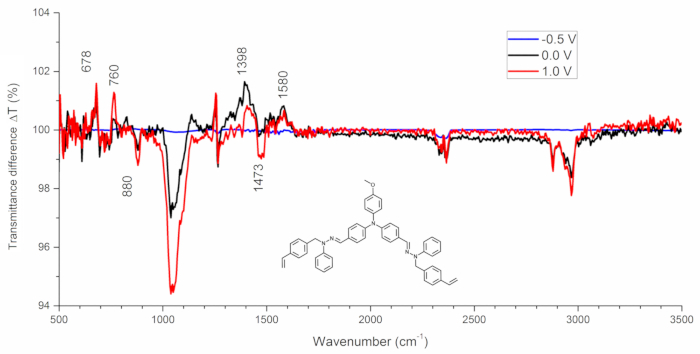

Figure 4 **: IR spectra of the monomer at various potentials applied** Please click here to view a larger version of this figure.

**Figure:**
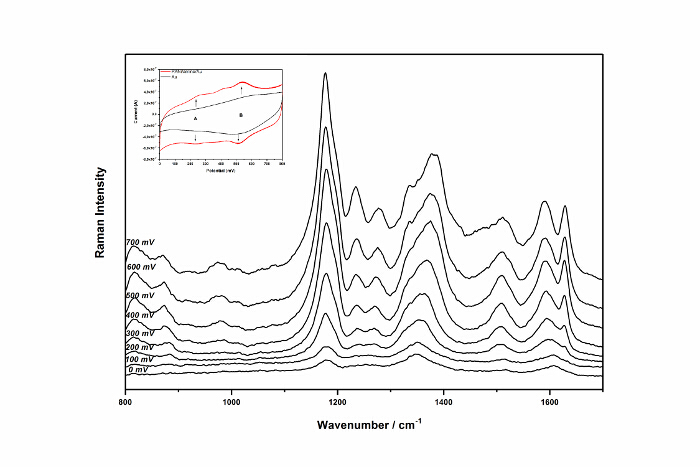

Figure 5 **: Raman spectra of polyaniline at a different potential; insert: CV curve recorded for the polyaniline film** Please click here to view a larger version of this figure.

## Discussion

Both IR and Raman techniques are recommended for the investigation of the structural changes occurring under applied potential and for the investigation of the products of the redox reaction. However, from the practical point of view, Raman spectroscopy is handier as an analytical tool in such experiments. Raman spectroelectrochemistry gives more possibilities, as it can be also applied to samples with nonpolar bonds. It has been therefore successfully used for the investigation of carbon materials, polymers, batteries, *etc*.^29^,^30^,^31^,^32^,^33^ Since the scattered light is measured substantially in Raman spectroscopy, there are generally no limits in the working electrode material or construction. Additionally, as used herein, incident light (UV-Vis-NIR) is poorly absorbed by the glass, which allows for the use of a standard electrochemical cell. The great advantage is also the possibility of conducting measurements outside the spectrometer through fiber optics. In order to register a Raman spectrum, the incident light needs to be properly focused on the sample. By focusing the light beam at different locations of the measuring cell, it can be decided if the changes in chemical composition occurring in the solution, *e.g*. near the electrode, or in the species adsorbed on the electrode surface are followed.

The use of Raman spectroscopy with an appropriate resolution also allows for the study of the profile of the solid samples, either on the surface or in its depths, also in the multi-layer structures.^34^,^35^,^36^,^37^ One can, therefore, get information about the surface topography, the distribution of different chemical species at the surface or in cross-section. Raman spectroelectrochemistry permits *in situ *tracking of the changes of all these features during redox processes and thus estimate the quality of the individual layers, the durability of the system during multiple oxidation/reduction cycles, or studying the diffusion in multilayer structures. The versatility of Raman spectroelectrochemistry lies in the fact that it can be used to examine both the electrochemical processes in a solution or solid state in a typical experimental cell or even test multilayer solid structures like LEDs, batteries, OPVs, *etc*.

The undoubted disadvantage of Raman spectroscopy and, thus also spectroelectrochemistry, is its limitation due to observed fluorescence, which often makes it impossible to analyze the spectrum. This phenomenon can be in some cases eliminated by changing the excitation wavelength or preliminary illumination - photo-bleaching.

## Disclosures

The authors have nothing to disclose.
